# Blood Pressure and Its Association with Gender, Body Mass Index, Smoking, and Family History among University Students

**DOI:** 10.1155/2018/4186496

**Published:** 2018-05-29

**Authors:** Hussein H. Alhawari, Sameeha Al-Shelleh, Hussam H. Alhawari, Aseel Al-Saudi, Dina Aljbour Al-Majali, Leen Al-Faris, Saif Aldeen AlRyalat

**Affiliations:** The University of Jordan, Queen Rania Street, Amman 11942, Jordan

## Abstract

Hypertension is one of the major risk factors associated with cardiovascular diseases. In this study, we will assess the frequency of hypertension among healthy university students and its association with gender, body mass index, smoking, and family history of both hypertension and cardiovascular diseases. We screened healthy university students ranging from 18 to 26 years of age. For each participant, we performed blood pressure measurements using a previously validated device and obtained demographic data, body mass index (BMI), smoking status, and family history of both hypertension and cardiovascular diseases. Out of the total number of 505 participants included in this study, 35.2% have blood pressure between 130/80 and 139/89, and 13.5% have blood pressure of more than 140/90. We found significant gender differences in both systolic pressure (*p* = 0.003) with mean difference = 18.08 mmHg (CI: 16.13 to 19.9) and diastolic pressure (*p* = 0.011) with mean difference = 3.6 mmHg (CI: 2.06 to 5.14), higher in males than in females. Upon comparing the mean difference in both systolic and diastolic blood pressure with BMI, we found significant differences in both systolic (*p* < 0.001) and diastolic (*p* = 0.002) blood pressure. We also found that smokers have significantly (*p* = 0.025) higher systolic blood pressure (mean difference = 4.2 mmHg, CI: 3.2 mmHg to 8.8 mmHg), but no significant difference for diastolic blood pressure (*p* = 0.386), compared to nonsmokers. First-degree family history of both hypertension and cardiovascular diseases affected systolic but not diastolic blood pressure. Taking into account the adverse short- and long-term effect of hypertension, we recommend adopting an awareness program highlighting the importance of screening blood pressure in young adolescent populations, keeping in mind that both high BMI and smoking are important modifiable factors.

## 1. Introduction

The Middle East is known to have a significantly higher percentage of younger populations compared to the west, with the prevalence of disabling cardiovascular diseases in those younger populations significantly higher compared to western populations [1]. Moreover, the healthier the younger populations, the higher the educational achievement and the more productive they are for their developing societies [2]. A previous study showed that, in developing countries, younger populations are poorly screened for diseases, especially those associated with long-term outcomes [3].

Hypertension is one of the major risk factors associated with cardiovascular diseases, and it is also a component of the metabolic syndrome [4]. Recently, the American Heart Association and the American College of Cardiology issued new guidelines to diagnose hypertension [5]. In these guidelines, they lowered the limit to diagnose stage 1 hypertension from 140 mmHg systolic and 90 mmHg diastolic to 130 mmHg systolic and 80 mmHg diastolic. Lowering the limit for hypertension diagnosis will increase the number of adolescents diagnosed as hypertensive. A recent study in the Philippines found a frequency of hypertension around 2.4% among university students (i.e., blood pressure > 140/90 mmHg), and 13.9% were considered prehypertensive (i.e., blood pressure > 130/80 mmHg) [6], but according to the new guidelines mentioned above, both groups are now considered hypertensive. In this study, we included healthy university students from healthcare faculties looking for the frequency of hypertension and the effects of gender, BMI, smoking, and family history of both hypertension and cardiovascular diseases.

## 2. Materials and Methods

This study was approved by our ethical committee and was conducted in concordance with the Declaration of Helsinki's latest report.

### 2.1. Participants

We recruited university students studying at the University of Jordan in healthcare faculties, including faculty of medicine, pharmacy, and nursing. We only included healthy students (i.e., for the purposes of this study, those not previously diagnosed with hypertension). Each participant involved in this study had a previous detailed checkup upon admission to the university; the checkup included a complete history and physical examination, in addition to complete blood count and urine analysis. We recruited participants via an announcement published in relevant social media websites. Each eligible participant was instructed not to take any stimulant before blood pressure measurement (e.g., coffee and tea) for at least one hour. We measured each participant's height, weight, and both systolic and diastolic blood pressure at the nephrology clinics. Body mass index (BMI) was calculated as weight in kilograms divided by height in meters squared (kg/m2).

### 2.2. Blood Pressure Measurement

We used an upper arm automated blood pressure device (an Omron 705IT (HEM-759-E)) for blood pressure measurement, a validated blood pressure device [7]. Three of the authors (A.A., D.A., and L.F.) were previously trained on blood pressure measurement, and they were instructed to rest the participant for 5 minutes prior to the measurement, support participant's arm, choose the correct cuff size, and place it over a bare arm. They measured blood pressure for each arm twice and recorded the mean for each arm, but we only included the higher mean reading for each participant.

### 2.3. Other Variables

We obtained both family history of either hypertension or cardiovascular diseases (either first degree, 2nd degree, or beyond) and smoking history (measured in pack-year).

### 2.4. Statistical Analysis

We used SPSS version 21.0 (Chicago, USA). We described our included sample using frequency (% proportion) for gender and mean (± standard deviation) for weight, height, systolic pressure, diastolic pressure, and BMI. We categorized BMI into the following groups: 18.5–24.9, 25–29.9, and 30–34.9, as shown in Table 4. We also categorized both systolic and diastolic blood pressure into the groups shown in Table 2. Due to the small number of participants in each group, we transformed smoking status into either a smoker (regardless of pack-year) or a nonsmoker.

We used Chi-square test followed by *Z*-test for proportions to analyze the relation between hypertension status (nonhypertensive, stage 1 hypertension, and stage 2 hypertension) and both gender and BMI category. We used independent sample *t*-test to analyze the mean difference in both systolic and diastolic blood pressure between each gender and between smoking statuses. We used one-way ANOVA to analyze the mean differences in both systolic and diastolic blood pressure for BMI and family history of both hypertension and cardiovascular diseases. We reported our data using mean difference and 95% confidence interval (CI), and we used a *p* value threshold of 0.05.

## 3. Results

A total of 505 participants were included in this study with a mean age of 21.2 years (±1.4, ranging from 18 to 26 years). They were 188 (37.2%) men and 317 (62.8%) women. Mean, standard deviation, minimum, and maximum for the included sample's weight, height, systolic BP, diastolic BP, and BMI are shown in Table 1. Out of the total 505 participants, the percentages of underweight, normal, overweight, and obese were 8.7%, 66.1%, 21.4%, and 3.8%, respectively.

For smoking, only 111 (22%) participants were smokers with a mean of 1.04 pack-years (±2.08). Family history of hypertension is present in 126 (25%), as 77 (15.3%) have a first-degree history and 49 (9.7%) have a second-degree family history of hypertension. Family history of cardiovascular diseases is present in 46 (9.1%), as 15 (3%) have a first-degree history and 31 (6.1%) have a second-degree family history of cardiovascular diseases.

Detailed frequencies for each hypertensive category are shown in Table 2. For systolic blood pressure, we found that 90 (17.8%) participants have blood pressure equal to or more than 130 mmHg, and 31 (6.1%) participants have blood pressure equal to or more than 140 mmHg. For diastolic blood pressure, we found that 229 (45.4%) participants have blood pressure equal to or more than 80 mmHg, and 53 (10.5%) participants have diastolic blood pressure equal to or more than 90 mmHg.

We found significant gender difference (*p* < 0.001) regarding hypertension, as 25.1% of males do not have hypertension (<130/80) compared to 74.9% of females. Moreover, we found significant difference (*p* < 0.001) between each BMI category and hypertension status. Details regarding both gender differences and the difference in each BMI category with hypertension status are found in Table 3.

We found significant gender differences in both systolic BP (*p* = 0.003) with mean difference = 18.08 mmHg (CI: 16.13 to 19.9) and diastolic BP (*p* = 0.011) with mean difference = 3.6 mmHg (CI: 2.06 to 5.14). Upon comparing the mean differences in both systolic and diastolic blood pressure with BMI, we found significant differences in both systolic (*p* < 0.001) and diastolic (*p* = 0.002) blood pressure. Details regarding these differences are shown in Table 4. For smoking status, we found that smokers have significantly (*p* = 0.025) high systolic blood pressure (mean difference = 4.2 mmHg, CI: 3.2 mmHg to 8.8 mmHg), but no significant difference for diastolic blood pressure (*p* = 0.386). Upon comparing the mean differences in both systolic and diastolic blood pressure among participants with (first or second degree) and without family history of hypertension, we only observed significant difference in mean systolic blood pressure (but not diastolic) in participants with first-degree family history of hypertension (but not in second degree), as systolic blood pressure for those with first-degree family history compared to those without is higher by a mean of 4.4 mmHg (CI: 0.4 mmHg to 8.3 mmHg). Family history of cardiovascular diseases has a similar effect as found in hypertension, as a difference in blood pressure is only found in systolic blood pressure upon comparing participants with first-degree history of cardiovascular diseases and those without (mean difference of 6.7 mmHg higher in those with a family history, CI: 1.6 mmHg to 15.1 mmHg).

## 4. Discussion

In this study, we found that if we were to apply the most recent threshold for the diagnosis of hypertension [5], 17.8% of our university students will be diagnosed with systolic hypertension (i.e., blood pressure > 130 mmHg) compared to 6.1% using the previous guidelines (i.e., blood pressure > 140 mmHg), and 45.4% of the students will be diagnosed with diastolic hypertension (i.e., blood pressure > 80 mmHg), compared to 10.5% using the previous guidelines (i.e., blood pressure > 90 mmHg). Moreover, we found significant gender differences in both systolic blood pressure with mean difference = 18.08 mmHg and diastolic blood pressure with mean difference = 3.6 mmHg, higher in males than in females.

BMI was also a major contributor to the high blood pressure in our university students, as the mean difference in systolic blood pressure between participants with BMI < 18.5 and 30–34.5 was 24.8 mmHg, and the mean difference in diastolic blood pressure between participants with BMI < 18.5 and 30–34.5 was 7.5 mmHg.

Previous studies showed that adolescents are not usually exposed to situations that require blood pressure measurements [3], despite the fact that several reports, including the present study, show that adolescents have relatively high rates of blood pressure, especially with the lower limits adopted recently for hypertension diagnosis [5], which were found mostly during studies [8]. Early detection of high blood pressure is usually difficult in adolescents, mostly due to underestimation for the long-term effect of high blood pressure and BMI and the relatively minor effect of these diseases on the well-being of adolescents compared to adults [9].

Generally, overweight and obese adolescents have more body fat and higher blood pressure than normal weight adolescents [8], and a strong relation between BMI and blood pressure is well established for both systolic and diastolic blood pressure [10]. This relation is also present for our population, with stronger effect of BMI on systolic blood pressure. Previous studies found high prevalence rates of cardiovascular risk factors, including components of metabolic syndrome, in undergraduate university students, with frequencies reaching 60% [11]. The high prevalence of metabolic syndrome components and the strong relation between BMI and blood pressure should direct awareness for the importance of lowering BMI, which will improve blood pressure, especially for university students.

The effect of smoking on blood pressure can be divided into an acute effect due to sympathetic nervous system stimulation and a chronic effect due to chronic cigarette smoking [12]. Acutely, smoking leads to an increase in both heart rate and blood pressure through a mechanism involving sympathetic nervous system stimulation [13]. The chronic effect of smoking is controversial; several studies found a decrease in blood pressure in chronic smokers, but these studies were either outdated [14, 15] or only involved women [16]. A larger study from a nationally representative sample showed a finding consistent with our study, which is an increase in systolic blood pressure without an effect on diastolic pressure [17]. This effect on systolic without diastolic can be explained by aortic stiffness caused by smoking [18]. Moreover, smoking was recently found to be associated with hypertensive heart disease [19].

The effect of having a family history of hypertension in the development of hypertension is already established and in concordance with this study, where having more than one member or having closer relative diagnosed increased the risk of having hypertension [20]. Previous reports showed that boys have higher blood pressure than girls significantly after the age of 16 [21]. Our results were in concordance with these reports, as male students have mean systolic blood pressure of 18.1 mmHg higher than female students and mean diastolic blood pressure of 3.6 mmHg higher than female students, keeping in mind that the minimum age for our included sample was 18 years. The higher blood pressure in males compared to females was also found in several other populations [8, 10].

After issuing the new guidelines jointly by the American Heart Association and American College of Cardiology [5], debate increased on the low threshold of hypertension diagnosis proposed. In this study, after showing a significant gender difference in blood pressure, a difference that might reach approximately 20 mmHg in systolic blood pressure, we believe that it is time to adopt a hypertension threshold based on gender, age, and other demographic variables. Larger studies also appreciated the difference in blood pressure based on the demographic variables [22].

### 4.1. Study Limitations

Despite the rigorous instructions given to ensure blood pressure measurement accuracy, this study has some limitations. Including other factors that might affect blood pressure (e.g., blood tests for cholesterol) will further clarify the relation between blood pressure and BMI. Future studies should investigate the long-term effects of having high blood pressure early during adolescent life.

## 5. Conclusion

We conclude with the existence of a high frequency of high blood pressure among university students, especially if we adopt the new limits for hypertension diagnosis. We also found a significant association between BMI and smoking with blood pressure, both of which are associated with higher blood pressure (only higher systolic BP for smokers), and both can be modified. Moreover, gender differences also should be considered, as boys have higher mean blood pressure compared to girls. Finally, having first-degree family history of hypertension or cardiovascular diseases has been shown to be associated with higher systolic blood pressure.

## Figures and Tables

**Table 1:** Mean, standard deviation, minimum, and maximum for the included sample's measurements.

	*N*	Minimum	Maximum	Median	Mean	Std. Deviation
Weight (Kg)	505	41	128	63	65.52	14.39
Height (m)	505	1.46	1.97	1.61	1.68	0.092
Systolic (mmHg)	505	83	159	115	116.71	13.58
Diastolic (mmHg)	505	42	103	79	78.80	8.69
BMI (Kg/m^2^)	505	15.04	36.09	22.67	23.0	3.55
Smoking (Packs/year)	505	0	11	0	1.04	2.08

**Table 2:** Detailed frequencies for each hypertensive category.

blood pressure	New hypertension limit	Old hypertension limit	Frequency (%)
<130/80	Not hypertensive	Not hypertensive	259 (51.3%)
130–139/80–89	Stage 1 hypertension	Pre-hypertensive	35.2 (35.2%)
>=140/90	Stage 2 hypertension	Stage 1 hypertension	68 (13.5%)

**Table 3:** Details regarding both gender differences and the difference in each BMI category with hypertension status.

	Gender		BMI category			
	Male (%)	Female (%)	<18.5	18.5–24.9	25–29.9	30–34.9
Hypertension Status						
Non-Hypertensive	65	194	30	184	44	1
	25.1%	74.9%	11.6%	71.0%	17.0%	0.4%
Stage 1 hypertension	74	104	14	109	44	11
	41.6%	58.4%	7.9%	61.2%	24.7%	6.2%
Stage 2 hypertension	49	19	1	42	20	5
	72.1%	27.9%	1.5%	61.8%	29.4%	7.4%

**Table 4:** Differences in mean systolic and diastolic blood pressure between normal BMI (reference) and other BMI categories. The “*p* value” indicates the significant level of the mean difference, as significant levels with *p* values < 0.05 are annotated with *∗*.

Blood pressure	Reference BMI (18.5–24.9)	Other BMI	Mean difference between reference and other BMI	*p* value	95% Confidence Interval	
					Lower Bound	Upper Bound
Systolic	18.5–24.9	<18.5	6.811^*∗*^	.004	1.63	11.99
		25–29.9	−8.494^*∗*^	.000	−12.07	−4.92
		30–34.9	−17.978^*∗*^	.000	−25.60	−10.36

Diastolic	18.5–24.9	<18.5	2.240	.364	−1.31	5.79
		25–29.9	−2.073	.130	−4.52	.38
		30–34.9	−5.226^*∗*^	.050	−10.44	−.01

## Data Availability

Data are available upon request.

## References

[1] Lopez A. D., Mathers C. D., Ezzati M., Jamison D. T., Murray C. J. (2006). Global and regional burden of disease and risk factors, 2001: systematic analysis of population health data. *The Lancet*.

[2] Koivusilta L., Rimpelä A., Rimpelä M. (1998). Health related lifestyle in adolescence predicts adult educational level: a longitudinal study from Finland. *Journal of Epidemiology and Community Health*.

[3] Magalhães M. G. A. P. D. A., Farah B. Q. U., Barros M. V. I. G. D., Ritti-Dias R. M. E. (2015). Previous blood pressure measurement and associated factors in student adolescents. *Einstein*.

[4] O'Neill S., O'Driscoll L. (2015). Metabolic syndrome: a closer look at the growing epidemic and its associated pathologies. *Obesity Reviews*.

[5] Nishimura R. A., Otto C. M., Bonow R. O. (2017). 2017 AHA/ACC focused update of the 2014 aha/acc guideline for the management of patients with valvular heart disease: a report of the american college of cardiology/american heart association task force on clinical practice guidelines. *Journal of the American College of Cardiology*.

[6] Pengpid S., Peltzer K., Ferrer A. J. G. (2014). Prehypertension and associated factors among university students in the Philippines. *International Journal of Adolescent Medicine and Health*.

[7] Coleman A., Freeman P., Steel S., Shennan A. (2006). Validation of the Omron 705IT (HEM-759-E) oscillometric blood pressure monitoring device according to the British Hypertension Society protocol. *Blood Pressure Monitoring*.

[8] Menacho A. A., Campos R. G., da Silva Y. M., Abella C. P., Miguel de Arruda M. D. (2014). Nutritional status and blood pressure in adolescent students. *Archivos Argentinos de Pediatria*.

[9] Mathew E., Shaikh R. B., Al-Sharbatti S., Muttappallymyalil J., Sreedharan J., Basha S. A. (2012). Self-rated health, BMI, blood pressure, and perceived health needs of first year students at a Middle-Eastern medical university. *Preventive Medicine*.

[10] Nwachukwu D. C., Nwagha U. I., Obikili E. N. (2010). Assessment of body mass index and blood pressure among university students in, Enugu, South East, Nigeria. *Nigerian Journal of Medicine*.

[11] Zea-Robles A. C., León-Ariza H. H., Botero-Rosas D. A., Afanador-Castañeda H. D., Pinzón-Bravo L. A. (2014). University students’ cardiovascular risk factors and their relationship with body composition. *Revista de Salud Pública*.

[12] Virdis A., Giannarelli C., Neves M. F., Taddei S., Ghiadoni L. (2010). Cigarette smoking and hypertension. *Current Pharmaceutical Design*.

[13] Grassi G., Seravalle G., Calhoun D. A. (1994). Mechanisms responsible for sympathetic activation by cigarette smoking in humans. *Circulation*.

[14] Seltzer C. C. (1974). Effect of smoking on blood pressure. *American Heart Journal*.

[15] Green M. S., Jucha E., Luz Y. (1986). Blood pressure in smokers and nonsmokers: epidemiologic findings. *American Heart Journal*.

[16] Masala G., Bendinelli B., Versari D. (2008). Anthropometric and dietary determinants of blood pressure in over 7000 mediterranean women: the european prospective investigation into cancer and nutrition-florence cohort. *Journal of Hypertension*.

[17] Primatesta P., Falaschetti E., Gupta S., Marmot M. G., Poulter N. R. (2001). Association between smoking and blood pressure evidence from the health survey for England. *Hypertension*.

[18] Laurent S., Cockcroft J., van Bortel L. (2006). Expert consensus document on arterial stiffness: methodological issues and clinical applications. *European Heart Journal*.

[19] Carter B. D., Abnet C. C., Feskanich D. (2015). Smoking and mortality—beyond established causes. *The New England Journal of Medicine*.

[20] Van Der Sande M. A. B., Walraven G. E. L., Milligan P. J. M. (2001). Family history: an opportunity for early interventions and improved control of hypertension, obesity and diabetes. *Bulletin of the World Health Organization*.

[21] Krzyzaniak A., Krzywińska-Wiewiorowska M., Stawińska-Witoszyńska B. (2009). Blood pressure references for Polish children and adolescents. *European Journal of Pediatrics*.

[22] Scheidt-Nave C., Kamtsiuris P., Göwald A. (2012). German health interview and examination survey for adults (DEGS)—design, objectives and implementation of the first data collection wave. *BMC Public Health*.

